# Delay-Bounded and Cost-Limited RSU Deployment in Urban Vehicular Ad Hoc Networks

**DOI:** 10.3390/s18092764

**Published:** 2018-08-22

**Authors:** Huanhuan Yang, Zongpu Jia, Guojun Xie

**Affiliations:** School of Computer Science and Technology, Henan Polytechnic University, Jiaozuo 454000, China; yanghh94@home.hpu.edu.cn (H.Y.); xiegj92@home.hpu.edu.cn (G.X.)

**Keywords:** binary differential evolution algorithm, opposite-based learning, delay-bounded, cost-limited, roadside unit (RSU) deployment, vehicular ad hoc network (VANET)

## Abstract

As an auxiliary facility, roadside units (RSUs) can well improve the shortcomings incurred by ad hoc networks and promote network performance in a vehicular ad hoc network (VANET). However, deploying a large number of RSUs will lead to high installation and maintenance costs. Therefore, trying to find the best locations is a key issue when deploying RSUs with the set delay and budget. In this paper, we study the delay-bounded and cost-limited RSU deployment (DBCL) problem in urban VANET. We prove it is non-deterministic polynomial-time hard (NP-hard), and a binary differential evolution scheme is proposed to maximize the number of roads covered by deploying RSUs. Opposite-based learning is introduced to initialize the first generation, and a binary differential mutation operator is designed to obtain binary coding. A random variable is added to the traditional crossover operator to increase population diversity. Also, a greedy-based individual reparation and promotion algorithm is adopted to repair infeasible solutions violating given constraints, and to gain optimal feasible solutions with the compromise of given limits. Moreover, after selection, a solution promotion algorithm is executed to promote the best solution found in generation. Simulation is performed on analog trajectories sets, and results show that our proposed algorithm has a higher road coverage ratio and lower packet loss compared with other schemes.

## 1. Introduction

As an emerging technology, the vehicular ad hoc network (VANET) plays a significant role in the transportation area, and gives hope to address some severe problems that have troubled us for many years in our society, such as higher incidences of traffic accidents, serious traffic jams, etc. To realize these goals, data should be efficiently transmitted among vehicles, infrastructures, and upper-layer systems [1,2]. Vehicles equipped with on-board units (OBUs) enabled to vehicle-to-vehicle (V2V) communications and roadside units (RSUs) placed on travel roads make vehicle-to-infrastructure (V2I) communications necessary in VANET.

Since VANET has some of the typical shortcomings of ad hoc networks, such as quickly changing topology, fast speed, fleeting connectivity, etc., V2V communications may have poor performance in the collection or dissemination of raw sensory data generated by vehicles. This further adds some challenges for the implementation of delay-sensitive applications in VANET. As an auxiliary communication facility in VANET, RSU can cope with dynamically changeable topologies caused by the rapid movement of vehicles and effectively solve VANET access problems, as well as improve communication quality between vehicle nodes [3]. However, deploying a large number of RSUs is impossible, because it not only brings with it unbearable cost, but is also influenced by many other factors [4]. Thus, it is of high importance that the best deploying locations in candidate cites within the bounded delay and budget are found.

Jo et al. [5] pointed out that RSU-based data delivery can be divided into inbound and outbound data delivery. Inbound data delivery refers to the process of message dissemination in RSU, while packets transfer from vehicles to RSUs in outbound data delivery. Here, we consider outbound data delivery in urban VANET, and suppose information flow is from vehicles to RSUs. This means the applications applied in our research are delay-sensitive. Given a bounded delay and a limited budget, our goal was to find near-optimal places to try and increase the number of roads covered by RSUs.

In this paper, we study the DBCL problem in urban VANET. After converting urban road maps into a weighted graph, we formulate this problem as a 0–1 variation Knapsack problem, and a binary differential evolution scheme is proposed to solve it. Improvement of the general differential evolution algorithm includes binary coding, opposite learning-based population initialization, binary differential mutation, and an improved binomial crossover. A greedy-based individual reparation and promotion algorithm is also adopted to convert a constrained optimization problem into an unconstrained optimization one, and the solution promotion algorithm is introduced to find optimization qualities of best solutions.

The rest of this paper is organized as follows: We review some previous studies in Section 2; in Section 3, we formulate the DBCL problem and give a 0–1 covering the matrix-solving algorithm; a binary differential evolution algorithm is shown in Section 4; Section 5 gives simulation results of the proposed algorithm; and in Section 6, we conclude this paper.

## 2. Related Work

In this section, we describe many state-of-the-art studies about delay-sensitive data delivery and RSU deployment in VANET. As delay-sensitive data delivery is our area of interest, it is important that we present some relevant works about this topic. Alkharasani et al. [6] focused on the problem of optimizing routing configuration parameters in V2V communications in VANET. They made a tradeoff between frequently changeable topology in VANET and Quality of Service requirements. Banani et al. [7] showed a novel approach to selecting important safety messages for verification in VANET. They considered the sender’s location, direction, and proximity, and the relative time taken between vehicles to decrease the number of useless verified messages. Izaparedes et al. [8] solved the data dissemination problem in road safety applications in VANET by adopting two game-theoretical mechanisms. Zhu et al. [9] studied the problem of delay-constraint data aggregation in VANET, and proposed centralized and distributed aTree algorithms to establish a data aggregation tree and allocate waiting times to each node in the tree. He et al. [10] investigated the data collection problem in VANET with fast-changing traffic. They formulated this as a scheduling optimization problem, and the optimal dynamic programming solution and genetic algorithm were proposed to solve it. Palazzi et al. [11] studied the problem of gathering data in certain geographic areas, and proposed the Delay-Bounded Vehicular Data Gathering protocol to appropriately adopt the data-muling strategy and multi-hop strategy in the gathering process. All the above-mentioned works were useful to our research on delay-bounded RSU placement in VANET.

When it comes to the RSU deployment problem, some common factors needed to consider in RSU deployment are data transmission delay, RSU coverage ratio, and the RSU deploying cost, etc. Lots of research has been done to optimize one or more of the above-mentioned factors.

Aslam et al. [12] proposed a balloon optimization method, which was used to place RSUs in highways. They imitated the dynamic expansion of balloons in a two-dimensional space, to find optimal locations so that the average reporting time of one vehicle to its nearby RSU when an interesting event happened was minimized. Similar to [12], Aslam et al. [13] researched the RSU optimal placement problem for a limited number in a given region. Two different schemes, i.e., the Binary Integer Programming (BIP) scheme and Balloon Expansion Heuristic (BEH) scheme were presented in the paper. The branch and bound approach was adopted to find the best locations in BIP, while the balloon expansion analogy was applied for finding near-optimal locations in BEH. Patil et al. [14] presented a two-stage Verona diagram-based algorithm in an urban area. They took packet loss and packet transmission delay into consideration, and their goal was to minimize these two criteria. However, the deployment locations obtained by this algorithm is perhaps not always applicable, as it considers neither private land nor the obstructions on it, such as rivers and buildings.

Trullols et al. [15] regarded infrastructure nodes as Dimensional Points (DPs) and solved the limited DPs deployment problem in urban environments. They considered two different cases, which included the number of vehicles connecting to DPs, and the number of covering vehicles and its covering times. Then, the greedy-based algorithm and subzone algorithm were applied to these two cases. Although the proposed algorithms are applicable to large-scale scenarios, the necessary vehicle mobility data unfortunately give rise to personal privacy issues, making it difficult to collect this type of data. Li et al. [16] proposed two types of RSUs: the cable-connected RSU (c-RSU) and wireless RSU (w-RSU), which have different communication radii and placing costs, as well as totally different delay-bounded coverage. Given a limited budget and delay, they could find optimal sites to maximize delay-bounded RSU coverage so as to add the number of vehicles received with broadcasting packets when any c-RSU starts to disseminate messages in a fixed region. Zhu et al. [17] designed a C street model and proposed a greedy-based polynomial (GBP) time approximation algorithm to search for the best candidate locations to deploy RSUs. For complex urban environments, they designed the Cue model and tried shifting strategy-based algorithm to solve the coverage problem in VANET. Mehar et al. [18] combined the Dijkstra algorithm with a genetic algorithm to minimize end-to-end delay and deploy costs for delay-sensitive applications, and an optimized roadside unit placement for delay-sensitive applications in vehicular networks (ODEL) was presented in that study. Chi et al. [19] provided an intersection-based algorithm to ascertain the number of RSUs and optimization places so as to offer higher connectivity between RSUs and to reduce deploying costs. The greedy algorithm, dynamic algorithm, and hybrid algorithm were presented in this paper. They also proved that duplicated areas could get minimized when solutions were calculated using these methods.

Patra et al. [20] studied RSU deployment in a one-dimensional highway. They viewed this problem as a complex decision problem with multiple criteria, and an analytic hierarchy process (AHP) was used to reduce the total cost of deploying RSUs with limited RSU-to-RSU delay. AHP is able to decompose the given problem into a hierarchy of goal, criteria, and alternatives, but it is hard to judge the local and global weight. Kim et al. [21] investigated how RSUs could be deployed in three different places, i.e., fixed locations, public transport with known routes but which are not controllable, and government vehicles which were entirely controllable and owned vehicle densities. The budgeted maximum coverage problem (BMCP) and budgeted maximum coverage problem with cardinality constraints (BMCP-CC) were studied, and an approximation algorithm was introduced to solve this problem. Nikookaran et al. [22] selected the total cost of capital expenditure (CAPEX) and operating expenditure (OPEX) as their goal when deploying RSU, and a history of traffic information was also needed. After integer linear program (ILP) formulation, a rounding procedure was done. Barrachina et al. [23] presented a density-based roadside unit deployment policy (D-RSU) to quickly disseminate emergency messages in a system with lower costs, and inverse proportion was used to deploy RSU.

There are also some factors which affected RSU placements, such as things like network throughput, etc. Wu et al. [24] took wireless interference, vehicle distribution, and vehicle speeds into account and finally obtained an integer linear programming model. Their goal was to get a larger aggregate throughput. Similar to [24], Malandrino et al. [25] focused on a content downloading system and formulated the problem into a maximum flow one after considering the channel contention and data transfer paradigm. Their goal was to maximize system throughput.

The main optimization objectives of the above studies were data transmission delay, RSU deploying cost, and throughput, etc. They provided methods which were sufficient in helping us to study the delay-bounded and cost-limited RSU deployment problem. Although some research focused on the RSU coverage ratio, only a few of them studied the coverage of sub-areas. In regard to the vehicles coverage problem, vehicle mobility data is key to designing a deployment algorithm—however, this type of data is either difficult to obtain, or violates personal privacy. Therefore, we converted the coverage of vehicles into a coverage of sub-areas, and sub-areas were mentioned to the divided sub-roads in a given area where a binary differential evolution scheme was proposed to find the best locations in candidate cites, so as to maximize the number of roads covered by RSUs. Also, the only information which was necessary to perform the proposed scheme were length, vehicle density, and average speed in each sub-road.

## 3. System Model

### 3.1. Problem Description

In this paper, we investigate how RSUs can be deployed in urban VANET. It is reasonable to place RSUs in intersections, or segments between intersections. However, because Trullols et al. [15] pointed out that the best location to deploy RSUs was at intersections, we thus selected intersections as candidate cites when deploying RSUs. Several definitions related to this problem were the following:

**Definition** **1.** *Urban Road Map: This can model as a weighted graph, G=(V,E). $V=\{I_{1},I_{2},\ldots I_{n}\}$ is the intersection set, |V|=n, $E=\{E_{1},E_{2},\ldots E_{n}\}$ is the segment set, and $D=\{D_{1},D_{2},\ldots D_{n}\}$ is the relevant distance set.*

**Definition** **2.** *Urban Road Topology: This can be represented as a weighted graph, $G_{1}=(V_{1},E_{1})$. In G=(V,E), if $D_{i}>R$, $E_{i}$ is then divided into a couple of sub-roads which establishes the graph $G_{1}=(V_{1},E_{1})$. $V_{1}=\{V,M\},E_{1}=\{e(i,j),\forall i,j,i\in V_{1},j\in V_{1}\}$, $M=\{m_{1},m_{2}\ldots m_{k}\}$ forms vertexes when divided E into segments. $\left|V_{1}\right|=N,\left|E_{1}\right|=m$, R is the communication radius of the vehicle and RSU.*

For each $e_{ij}\in E_{1}$, we assume $e(i,j)=\{l_{ij},\rho_{ij},v_{ij}\}$, $l_{ij}$, $\rho_{ij}$, $v_{ij}$ are the Euclidean distance, vehicle density, and average speed of e(i,j), respectively. Then, the expected data transmission time [16,26,27,28] in e(i,j) can be computed using Formula (1), which is also a weight of $e_{ij}\in E_{1}$.

(1) $$t(i,j)=(1-e^{-R\cdot \rho_{ij}})\cdot \frac{l_{ij}\cdot t_{hop}}{R}+e^{{}^{-R\cdot \rho_{ij}}}\cdot \frac{l_{ij}}{v_{ij}}$$

**Figure 1:**
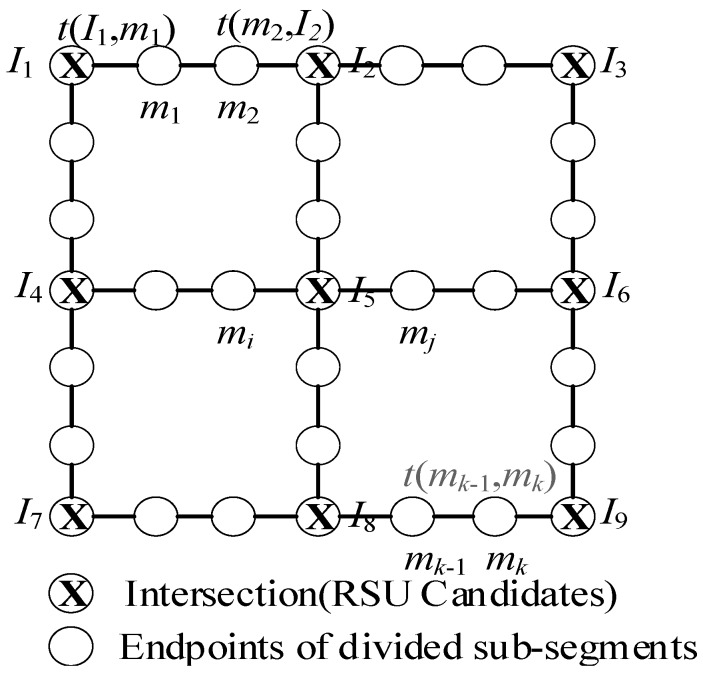
Urban road topology.

In Formula (1), $t_{hop}$ (Section 3.3) is one hop transmission time of packets. Figure 1 gives the urban road topology when *n* = 9. Notations used in this paper are summarized in Table 1.

### 3.2. DBCL Problem

We consider the delay-bounded and cost-limited RSU deployment problem in urban VANET. Our goal was to deploy RSUs within the given budget to maximize the number of segments able to transmit data to RSUs before τ.

**Definition** **3.** *The delay-bounded and cost-limited RSU deployment problem (DBCL): Given $t_{j}(1\leq j\leq m)$, which is the minimum transmission time from vehicles in e(i,j) to RSU. Then the DBCL problem can formulate as Formula (2), where $x_{i}$ and $y_{j}$ are 0–1 variables. For each e_ij_ ∈ E_1_, $y_{j}=1$, if $t_{j}\leq \tau$, and if $I_{i}$ is selected to deploy RSU, we set $x_{i}=1$.*

(2) $$\begin{array}{l} obj:\;\max\sum_{j=1\;}^{m}y_{j}\\ s.t.:\;\sum_{i=1}^{n}W_{i}\cdot x_{i}\leq C;\;y_{j}\in \{0,1\};\;x_{i}\in \{0,1\} \end{array}$$

**Theorem** **1.** *DBCL problem is a NP-hard problem.*

**Proof.**  To prove that the DBCL problem is NP-hard, a 0–1 Knapsack problem was introduced. It can be described as: Given *n* objects, their corresponding weights $W=\{w_{1},w_{2},\ldots w_{n}\}$, values $P=\{p_{1},p_{2},\ldots p_{m}\text{\}}$, and limited weight capacity C, the goal is to find a packing method so that objects packed in Knapsack can have a maximum value where their total weight does not exceed C. Since the 0–1 Knapsack problem is a NP-hard problem, we can convert the DBCL problem into a variation of the 0–1 Knapsack problem, where Theorem 1 can thereby be proved. □

**Definition** **4.** *τ-Limited 0–1 Knapsack Problem (τ-LKP). When $I_{i}(1\leq i\leq n)$ is merely selected for deploying RSU, subsets $P_{i}$ of $E_{1}$ mean that segments can transmit data to RSU at $I_{i}$ in τ. Then $P_{i}$ can be regarded as an object value and its deployment costs can be regarded as object weights. Therefore, the τ-LKP problem can be formalized as Formula (3).*

(3) $$\begin{array}{l} obj:\;\max\;\cup (\sum_{i=1\;}^{n}P_{i}\cdot x_{i})\\ s.t.:\;\sum_{i=1}^{n}W_{i}\cdot x_{i}\leq C;\;x_{i}\in \{0,1\} \end{array}$$

Therefore, the DBCL problem is a NP-hard problem.

### 3.3. Delay-Bounded Coverage

**Definition** **5.** *One-hop transmission time: If the distance from e(i,j) to RSU i∈V is lower than the communication radius of the vehicles and RSUs, given the data rate s, then the one-hop transmission time for a message of size $p_{size}$ from e(i,j) to RSU i∈V can be calculated using Formula (4).*

(4) $$t_{hop\;}=\frac{p_{size}}{s}$$

**Definition** **6.** *0–1 covering matrix. We define a m×n matrix T, which is given in Formula (5).*

(5) $$T_{ji}=\left\{ \begin{array}{l} 1,\;t_{ji}\leq \tau \\ 0\;,Otherwise \end{array}\right.$$

If we assume the distance from e(i,j) to RSU i∈V is $d_{ji}$, the minimum transmission time from vehicles in e(i,j) to RSU i∈V is $t_{ji}$, and $t_{ji}$ can then be computed using Formula (6).

(6) $$t_{ji\;}=\left\{ \begin{array}{ll} t_{hop}=\frac{p_{size}}{s}, & 0\leq d_{ji}\leq R\\ \min\{disk[i],disk[j]\}+t(i,j), & d_{ji}>R \end{array}\right.$$

In Formula (6), i,jare endpoints of e(i,j), while disk[i] and disk[j] are the minimum transmission times from i,j to RSU at $I_{i}$. If $d_{ji}$ is smaller than the communication radius, it means e(i,j) is directly connected with RSU at i∈V and $t_{ji}$ is equal to $t_{hop}$. Otherwise, the shortest path [16,18,27] (the minimum transmission time) is adopted to calculate $t_{ji}$ from $V_{1}$ to $I_{i}$ in $G_{1}=(V_{1},E_{1})$ through the Dijkstra algorithm [29]. t(i,j) is the expected data transmission time in e(i,j), and can be obtained via Formula (1) in Section 3.1.

Algorithm 1 shows how to get the 0–1 covering matrix in detail. An array disk is defined in Line 1 to record the minimum transmission time from all vertexes to $I_{i}$ in $G_{1}=(V_{1},E_{1})$. For any vertex $I_{i}$ in $G_{1}=(V_{1},E_{1})$, the minimum transmission time from $I_{i}$ to other vertexes is computed through the Dijkstra algorithm in Line 3. In matrix T(m×n), the i-th column ($T_{ji}(1\leq j\leq m,1\leq i\leq n)$) is obtained in Lines 4–8. We first compute $t_{ji}$ through Formula (6) in Line 5, then compare it with the bounded delay τ. If it satisfies $t_{ji}\leq \tau$, we set $T_{ji}=1$ (Line 6), otherwise we set $T_{ji}=0$ (Line 7). After Lines 1–9 have been performed, the 0–1 covering matrix can be formed.

**Algorithm 1.** Computing the 0–1 covering matrix.
**Input:**
$V=\{I_{1},I_{2},\ldots I_{n}\}$
**,**
$G_{1}=(V_{1},E_{1})$
**, delay**
τ

**Output:**
T(m×n)

**1: Double dist[N];**

**2: For int**
i=1
**to**
n
**3: Dijkstra (**$G_{1}$**, dist,**$I_{i}$);
**4: For int**
j=1
**to**
m

**5: Compute $t_{ji}$ according to Formula (6);**
**6: If (**$t_{ji}\leq \tau$**)**$T_{ji}=1$;**7: Else if (**$t_{ji}>\tau$**)**$T_{ji}=0$;
**8: End for**

**9: End for**


After the matrix $T_{m\times n}$ is obtained, the τ-covered sub-roads set $P_{i}$ can be expressed as Formula (7).

(7) $$P_{i}=\cup \sum_{j=1\;}^{m}\left(T_{ji}=1\right)$$

**Theorem** **2.** *The time complexity of Algorithm 1 is $O[n\times (N^{2}+m)]$.*

**Proof.**  Because the number of RSU candidate locations is n, the algorithm needs to be done n times. Since the time complexity of using the Dijkstra algorithm to compute the minimum transmission time from all vertexes to $I_{i}$ in $G_{1}=(V_{1},E_{1})$is $O(N^{2})$, the time complexity of calculating the minimum transmission time from $e_{ij}\in E_{1}$ to RSU at $I_{i}$ is O(m). In other words, the time complexity of each computation is $O(N^{2}+m)$—thus, the total running time is $O[n\times (N^{2}+m)]$. □

## 4. Binary Differential Evolution-Based RSU Deployment

In Section 3.2, we converted the DBCL problem into a τ-LKP problem. The aim of the problem was to determine the objects packed to Knapsack so that their total value was maximized and total weight was limited by the capacity. An n-dimensional vector x was introduced, which satisfied $x=\{x_{1},x_{2},\ldots ,x_{n}\}$, $x_{i}=1$ if the i-th object was packing, and vice versa. It can be seen that x is the solution vector of the τ-LKP problem.

Based on this, we propose the binary differential evolution-based RSU deployment scheme (BDERD). Unlike the general differential evolution algorithm, BDERD can solve combinatorial optimization problems in discrete solution spaces. It includes multiple operations, such as individual coding, population initialization, mutation operation, crossover operation, and selection operation.

### 4.1. Population Initialization

Individuals in the population are coded using the binary numbers 0 and 1. When an object is put into the Knapsack, it is coded 1, otherwise it is coded 0, after which the solution vector $x=\{x_{1},x_{2},\ldots ,x_{n}\}$ is formed. The aim of opposition-based learning [30], proposed by Tizhoosh in 2005, is to evaluate reverse solutions while assessing feasible solutions, and then to add the best solutions to the population.

**Definition** **7.** *Opposite Point (OP): For a point $P=\{x_{1},x_{2},\ldots ,x_{n}\}$ in an n-dimensional space, its opposite point $P^{\prime }=\{x_{1}{}^{\prime }$$x_{2}{}^{\prime },\ldots ,x_{n}{}^{\prime }\}$ is defined as Formula (8). Where $x_{1},x_{2},\ldots ,x_{n}\in R$ and $x_{i}\in [a_{i},b_{i}]$,*

(8) $$x_{i}{\;}^{\prime }=a_{i}+b_{i}-x_{i}$$

**Definition** **8.** *Opposite-based Learning (OBL): Wang et al. [31] summarized four types of generalized opposite-based learning models. They can be expressed as Formula (9), where k is a real number, and a and b are upper and lower bounds of the search space. If we set k= 0, 1/2, 1, and 2, we can obtain some solutions—namely, a symmetry-based general opposite learning model, a region symmetry-based general opposite learning model, a general opposite learning model, and a randomized general opposite learning model, respectively.*

(9) $$x^{*}=k(a+b)-x\;$$

The idea of using opposite-based learning was adopted to generate an initial population. Algorithm 2 fully demonstrates this process of generating an initial population. We first used the Bernoulli process to generate n-dimensional random vectors as an alternative population in Lines 2–7. Specifically, for any n-dimensional individual $x^{i}(1\leq i\leq NP)$ in the initial population (Lines 1–2), a uniformly distributed random number in [0,1] was generated in Line 3. Then we compared it with the probability p, and if it satisfied u≤p in the j-th dimension, we set $x^{i}(j)=1$ (Lines 4–5), otherwise $x^{i}(j)=0$ (Line 6). Here, we set p=0.5 to balance out the randomness of generating individual $x^{i}$. Once $x^{i}$ had been constructed, its opposite solution $x^{i\prime }$ was calculated by using Formula (9) in Line 8, and we set k=1 in Formula (9). So far, NP generated solutions and NP opposite solutions had existed in the alternative population. Then we computed the fitness of all vectors in this population in Line 10, and selected the former NP vectors as the initial population in Line 11. In Line 12, the optional solution $x^{*}$, and its fitness $B=f(x^{*})$, was obtained in the initial population.

**Algorithm 2.** Population Initialization.
**Input: population size**
NP
**, fitness function**
f(x)

**Output: initial population**
$P=\{x^{1},x^{2},\ldots x^{M}\}$
**1: For int**i=1**to**NP;
**2: For int**
j=1
**to**
n

**3: Generate a uniformly distributed random**
u
**in [0, 1];**

**4: If**
u≤p

**5:**
$x^{i}(j)=1;$

**6: Else**
$x^{i}(j)=0;$

**7: End for**

**8: Calculate**
$x^{i\prime }$
**through Formula (9);**

**9: End for**

**10: Compute fitness of all vectors in alternative population;**
**11: Select the former**NP vectors as initial population;
**12: Compute the optional solution**
$x^{*}$
**and its fitness**
$B=f(x^{*})$
**in initial population**


### 4.2. Individual Reparation and Promotion

During the implementation of BDERD, some vectors may violate the constraint in Section 3.2—or in other words, the total weight of objects packed in the Knapsack may go beyond C. For these kinds of constrained optimization problems, the most commonly-used method to transform it into an unconstrained optimization problem is to use the penalty function—however, it is difficult to determine what the penalty factor is. Thus, we used the greedy-based individual reparation and promotion algorithm to turn the constrained optimization problem into an unconstrained one. The basic idea was to repair infeasible solutions and to promote quality of feasible solutions. Therefore, the fitness function of an individual in BDERD is equal to the objective function of τ-LKP, and this can be symbolized as Formula (10).

(10) $$f(x)=\sum_{k=1\;}^{n}x^{i}(k)\cdot P_{k}$$

The greedy-based individual reparation and promotion algorithm is shown in Algorithm 3. Prior to this, we defined the value-to-weight ratio of each object as Formula (11):

(11) $$\rho_{i}=\frac{P_{i}\;}{W_{i}}$$

After this, a descending-number array H={1,2,…,n] corresponding to each object was obtained according to the value-to-weight ratio. Methods used to repair infeasible solutions can be divided into the first reparation and the second reparation, in accordance with two different repairing orders [32]. Here, we used the second reparation method, i.e., the descending reparation order to repair individuals. Algorithm 3 demonstrates this process.

**Algorithm 3.** Individual reparation and promotion.
**Input: solution vector**
$x^{j}=\{x^{j}(1),x^{j}(2),\ldots ,x^{j}(n)\}$

**Output: repaired and promoted solution vector**
$x^{j}=\{x^{j}(1),x^{j}(2),\ldots ,x^{j}(n)\}$

**1: For int**
k=1
**to**
n

**2: Compute value-to-weight of *I_k_*; end for**

**3: Compute H={1,2,…,n] according to value-to-weight**

**4: If ($\sum_{k=1}^{n}x^{i}(k)\cdot W_{k}>C$)**

**5: Int $Al_{value}=0$;**

**6: For int**
k=1
**to**
n

**7: If ($\left(x^{i}(k)==1)\&\&(Al_{value}+W_{H[k]}>C\right)$)**

**8: $x^{i}(j)=0$;**

**9: Else**
$Al_{value}=Al_{value}+W_{H[k]};$

**10: End for**

**11: End if**

**12: Else if ($\sum_{k=1}^{n}x^{i}(k)\cdot W_{k}\leq C$)**

**13: Int**
$Al_{value}=\sum_{k=1}^{n}x^{i}(k)\cdot W_{k};$

**14: For int**
k=1
**to**
n

**15: If ($\left(x^{i}(k)==0)\&\&(Al_{value}+W_{H[k]}\leq C\right)$)**

**16:**
$x^{i}(j)=1$
**;**
$Al_{value}=Al_{value}+W_{H[k]};$

**17: End for**

**18: End else**


Algorithm 3 starts with computing the value-to-weight rate for each candidate location (intersection) in the road network in Lines 1–2. Then, we defined an array H recording the index number of each candidate location, and sorted it according to the relevant value-to-weight rate in a descending order in Line 3. For each individual in the population, we computed the total weight of objects packed in the Knapsack and compared it with the capacity C. If it exceeded C, Lines 4–11 were performed to repair the infeasible solution. Otherwise, Lines 12–18 were performed to promote the quality of the feasible solutions. In cases where the total weight exceeded C (Line 4), we first defined a variable $Al_{value}$, which was the total valid weight of related objects packed into the Knapsack and set it to equal 0 in Line 5. Then, for each n-dimension in vector $x^{i}$, if the *k*-th dimension was equal to 1 and the sum of the weight of object k plus the total valid weight $Al_{value}$ is more than C, we change the value of the *k*-th dimension in $x^{i}$ to 0 in Lines 7–8. If not, the total valid weight $Al_{value}$ can be added to the weight of object *k* in Line 9. This process (Lines 4–11) is continued until all dimensions in the vector have been judged. In cases where the total weight does not exceed C (Line 12), $Al_{value}$ is also defined and calculated as the total valid weight of related objects packed in Knapsack, through Line 13. Then, for each *n*-dimension in vector $x^{i}$, if the *k*-th dimensional is equal to 0 and the sum of the weight of object k plus the total valid weight $Al_{value}$ is less than C, the value of the *k*-th dimensional in $x^{i}$ is changed to 1, and the total valid weight $Al_{value}$ is added to the weight of object *k* in Lines 15–16. We repeatedly executed Lines 12–18 until all the dimensions in the vector had been judged.

### 4.3. Mutation, Crossover, and Selection

Scholars have proposed different mutation strategies (DE/rand/1, DE/best/1, DE/rand-to-best/1, etc.) in general differential evolution algorithms to perform mutation operations in continuous solution spaces. However, individuals in BDERD are coded by binary numbers, which cannot directly use these mutation strategies. Thus, a binary differential mutation strategy was designed for vectors in discrete solution spaces, as shown by Formula (12).

(12) $$v^{i}=(x^{*}+x^{r1\;}+x^{r2}-1)+{\left(-1\right)}^{x^{*}}\cdot \left|x^{r1}-x^{r2}\right|$$

In Formula (12), $v^{i}$is a mutation individual, $x^{*}$ is the best individual in the population, r1 and r2 are two random integers in [1,NP], and r1≠r2. Once a dimension in $v^{i}$ crosses the boundary, the truncation method in Formula (13) is used to process this transboundary dimension.

(13) $$v^{i}=\left\{ \begin{array}{ll} 0 & v^{i}<0\\ 1 & v^{i}>1 \end{array}\;\right.$$

The designed binary differential mutation strategy can retain same coding in individuals to avoid losing some good code structures and to maintain population diversity. Table 2 further demonstrates the functions of this strategy.

The crossover operator used in BDERD is called the binomial operator, where as different from the traditional binomial operator, a random disturbance is added to enhance population diversity in Formula (14). Given the crossover rate $C_{R}$, random rate $C_{new}$, mutation vector *v^i^*, and population vector $x^{i}$, the trial vector $u^{i}$ is then equal to:

(14) $$u^{i}(j)=\left\{ \begin{array}{l} v^{i}(j)\;,rand\leq C_{R}\\ x^{i}(j),\;C_{R}<rand\leq C_{new\;}\\ rand\mathrm{int}[0,1],\;rand>C_{new} \end{array}\right.$$

$C_{R}\in (0,1]$ is the crossover rate and in a range of (0.4, 1) [33], $C_{new}$is the probability of generating a new random number, randis the uniform random variable in [0, 1], and randint[0,1] is a random number between 0 and 1. $v^{i}(j)$, $x^{i}(j)$, and $u^{i}(j)$ are the j-th dimension of mutation vector $v^{i}$, population vector $x^{i}$, and trail vector $u^{i}$.

The idea of the “survival of the fittest” was applied to select optional vectors between population vector $x^{i}$ and trail vector $u^{i}$, according to their fitness after the crossover was finished. For a maximum problem, vectors with higher fitness will remain in the next generation to improve convergence speed. The common selection operator is:

(15) $$x^{i}(G+1)=\left\{ \begin{array}{l} u^{i},\;f(u^{i})>f(x^{i})\\ x^{i},\;Otherwise \end{array}\;\right.$$

where $x^{i}(G+1)$ is a vector in the next generation, and$f(x^{i})$ and $f(u^{i})$ are the fitness of population vector $x^{i}$ and trail vector $u^{i}$ in the current generation.

### 4.4. Solution Promotion

Solution promotion plays an important role in obtaining the best vectors in a population. After mutation, crossover, and selection, another best vector is able to be obtained, $x^{s}$. Defining a variant set T, its elements can be given by $T=\{i\in \{1,2,\ldots n\},x^{s}\neq x^{*}\}$, i.e., members when $x^{*}$ is not equal to $x^{s}$.

Algorithm 4 shows the solution promotion algorithm. Its core idea is to gradually turn over the elements in $x^{s}$ which satisfy $x^{s}(i)\neq x^{*}(i)$, and to make a comparison between the fitness of $x^{*}$ and the new $x^{s}$ until T is empty. In Line 2, we changed the elements in $x^{s}$ which satisfied $x^{s}(i)\neq x^{*}(i)$, and computed the fitness of new $x^{s}$ in Line 3. Then we compared the fitness of new $x^{s}$ and that of $x^{*}$, and if the former was bigger than the latter, we let $x^{*}$ equal to the new $x^{s}$ in Lines 4–5; otherwise, we dropped this element from the differential set T in Lines 6–7. Lines 1–8 were repeatedly implemented until T became empty. Finally, after these processes, the promoted $x^{*}$ will result (Line 9).

**Algorithm 4.** Solution promotion algorithm.
**Input:**
$x^{*}$
**,**
$x^{s}$
**, differential set**
T

**Output:**
$x^{*}$

**1: While**
T=ϕ

**2: Change**
$x^{s}(i)$
**in**
$x^{s}$
**as $1-x^{s}(i)$;**

**3: Compute**
$f(x^{s})$
**,**
$x^{s}=\{x^{s}(1),x^{s}(2),\ldots ,1-x^{s}(i),\ldots ,x^{s}(n)\};$

**4: If ($f(x^{s})>B=f(x^{*})$)**

**5: $x^{*}=x^{s}$;**

**6: Else if ($f(x^{s})\leq B=f(x^{*})$)**

**7: $T=T-\{x^{s}(j)\}$;**

**8: End while**

**9: Return $x^{*}$;**


### 4.5. Flow of Binary Differential Evolution Scheme

**Figure 2:**
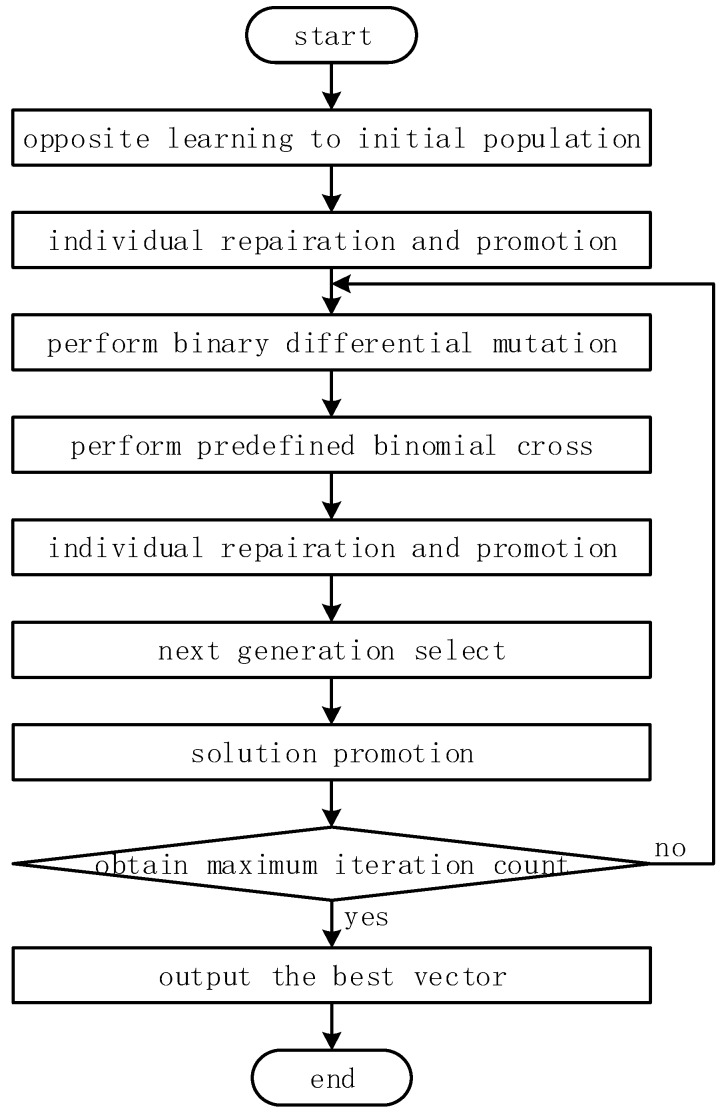
Flow chart of the binary differential evolution scheme.

On the basis of the aforementioned design, Figure 2 shows the flow chart of the binary differential evolution scheme. General steps of this scheme are outlined as the following:

Step 1: Using the Bernoulli process to generate n-dimensional random vectors, and adding their opposite vectors to comprise an alternative population, former NP vectors are taken as the initial population after calculating their fitness, based on the fitness function.
Step 2: The greedy-based individual reparation and promotion algorithm is done to repair infeasible vectors and promote quality of feasible vectors, Then, the best vector $x^{*}$ and its fitness as $B=f(x^{*})$ is remarked in the initial population.
Step 3: Mutation vectors are obtained after doing the binary differential mutation strategy.
Step 4: When the improved crossover operator is performed, the greedy-based individual reparation and promotion algorithm is re-executed for content limitations.
Step 5: The next generation is selected on the basis of the selection operator, then the best vector $x^{s}$, as well as its fitness $f(x^{s})$, is obtained in the next generation.
Step 6: Perform the solution promotion algorithm to update the best vector in the generation.
Step 7: Repeat Step 3 to Step 6 until the maximum iteration count is reached.

## 5. Simulations

In this section, we outline the simulation results of our BDERD scheme to other well-known RSU deployment schemes in the synthetic scenery (Section 5.2.) and realistic scenery (Section 5.3.). The performance metrics and specific details of these schemes are shown in Section 5.1. In order to promote the accuracy of the simulation results, all data obtained were computed by taking the average value of the 20 trial runs.

### 5.1. Performance Measures

In this study, we used four state-of-the-art RSU deployment schemes to evaluate our proposed BDERD scheme. They can be portrayed as follows:

**The genetic algorithm deployment scheme (GA)** [18]. Similar to our BDERD scheme, GA also involves individual encoding, initialization, selection, crossover, and mutation operations. The deployment locations of RSUs are selected when they have a high coverage and low cost after the scheme has been performed for the defined generations.

**The greedy-based budget maximum coverage problem (BMCP-g)** [21]**.** The BMCP-g scheme applies the (1–*1/e*)-approximation greedy algorithm to find the best locations to deploy RSUs. The solutions outputted by this scheme are candidate sites that have a high weight and cost not exceeding *L*.

**The hot deployment scheme (Hot)** and **uniform deployment scheme (Uniform)** [24]. These are widely-used deployment schemes when deploying RSUs in VANET. RSUs are placed in hot spot sites in the Hot scheme, while they are spread out in a fixed area in the Uniform scheme.

To analyze the performance of these deployment schemes, four different metrics are used:

Road coverage ratio: It is vital to measure the effectiveness of deployment schemes, and it is divided by the number of valid coverage sub-roads to all sub-roads in the graph. The number of valid coverage sub-roads is calculated by subtracting duplicated sub-roads from all sub-roads.

Packet loss ratio: This is divided by the number of packet loss to all packets. The number of losing packets is computed by subtracting the number of packets successfully transmitted in the delay from all packets in the deploying area.

Number of RSUs: This is directly decided by the budget, and is limited by two constant values—one being divided by the budget to the minimum deployment cost of the candidates, and the other being divided by the maximum.

Average transmission time: This has a significant effect on outbound data delivery, especially when some applications are sensitive to delay. It is divided by the sum of the transmission time for segments which can transmit its data to the covering RSUs within delay to the number of these segments. For some sub-roads covered by multiple RSUs, vehicles in these roads can transfer their packets to any of the covering RSUs in theory, and there are multiple transmission times within the existing bounded delays. In this case, we take the average transmission time of these times as the vehicle transmission time.

### 5.2. Synthetic Simulation

**Figure 3:**
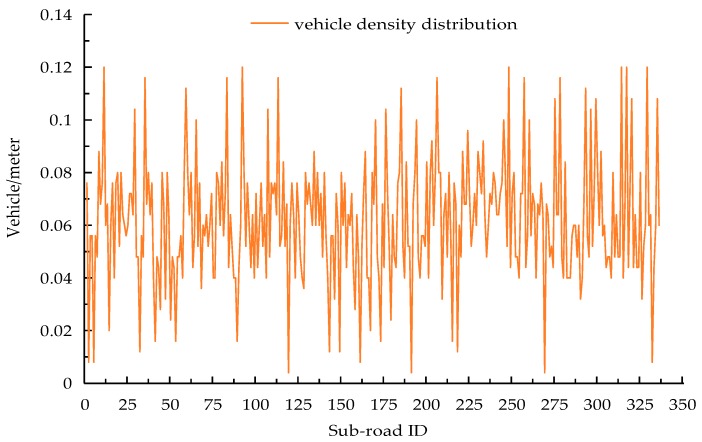
Vehicle density distribution in synthetic simulation.

In order to assess the performance of the above-mentioned schemes, we firstly modelled a city road map and converted it into a weighted graph to get the 0–1 covering matrix. We then ran these five schemes on this matrix. The deployment area was c×c, which contained $c^{2}$ intersections, and each segment between two adjacent intersections was divided into three sub-roads. The length of each sub-road was equal to the communication radius of RSU, i.e., 250 m, meaning the size of the deployable area was 750(c−1)×750(c−1), e.g., a 5×5 region means there are 25 junctions and 120 sub-roads in the deployable area, and its size is 3 km×3 km. The bounded delay for data transmission in each sub-road was set to 4 s, and the total cost for RSU deployment was 200. We supposed the data rate for transmission was 3 Mb/s, and vehicles moved through each sub-road at a varying average speed of 15–50 km/h. They also generated their own 1 KB data packet during the bounded delay. The vehicle density of each sub-road was not uniformly distributed, and the sub-roads which were closer to the intersections had a higher density. Figure 3 fully demonstrates the detail of the traffic scenery in each sub-road. When we performed our BDERD scheme, we set the crossover rate to $C_{R}=0.9$ [34], the random rate to $C_{new}=0.6$, the population size to 50, and the generation to 100. When performing the GA scheme, we set the crossover rate to 0.6, the mutation rate to 0.1, the population size to 100, and the generation to 200. We studied how the bounded delay, the total budget, and the size of the area could affect the aforementioned performance metrics.

#### 5.2.1. Effect of Bounded Delay

**Figure 4:**
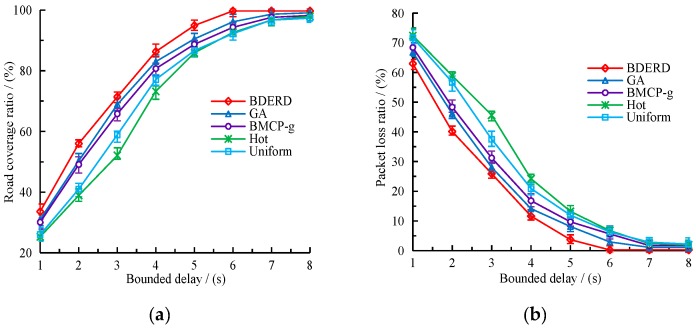
(**a**) Road coverage ratio versus bounded delay; (**b**) packet loss ratio versus bounded delay.

**Figure 5:**
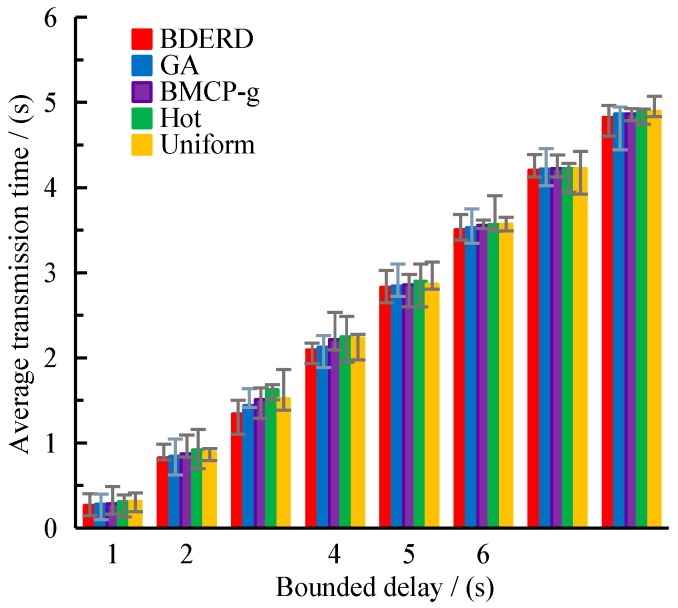
Average transmission time versus the bounded delay.

In this section, we investigate the impact of the bounded delay on the varying schemes’ performance. The deployment area was 8×8, the total budget was 200, and the bounded delay varied from 1 s to 8 s, with a 1 s gap. Figure 4a,b depict the effect of the bounded delay on the road coverage ratio and packet loss ratio. From the figures, we can draw a conclusion that as the bounded delay is prolonged, the road coverage ratio gradually grows, whereas the packet loss ratio gradually drops—however, both become stable in the end. It is no doubt that an increasing bounded delay also increases the number of roads covered by RSUs, since more time will be given to transmit packets. The BDERD scheme can find the best deployment locations with a global consideration of coverage sub-roads, deploy cost, and adopt a variety of methods to search the near-optimal solutions. This creates a higher road coverage ratio and a lower packet loss ratio. Figure 5 presents the changes of average transmission time with the bounded delay.

We can see from these figures that the average transmission time gradually increases as the bounded delay becomes longer. In fact, it remains stable when the bounded delay has exceeded the largest carry-and-forward data transmission time in the given road network. It is reasonable that a gradually increasing bounded delay allows more vehicles to transmit their data based on the carrying strategy instead of the forward strategy, which would further enlarge the average data transmission time. Although the differences between the varying schemes are slight, we can still find that the BDERD scheme has a smaller average transmission time due to the best deployment sites it was able to find.

#### 5.2.2. Effect of Total Budget

**Figure 6:**
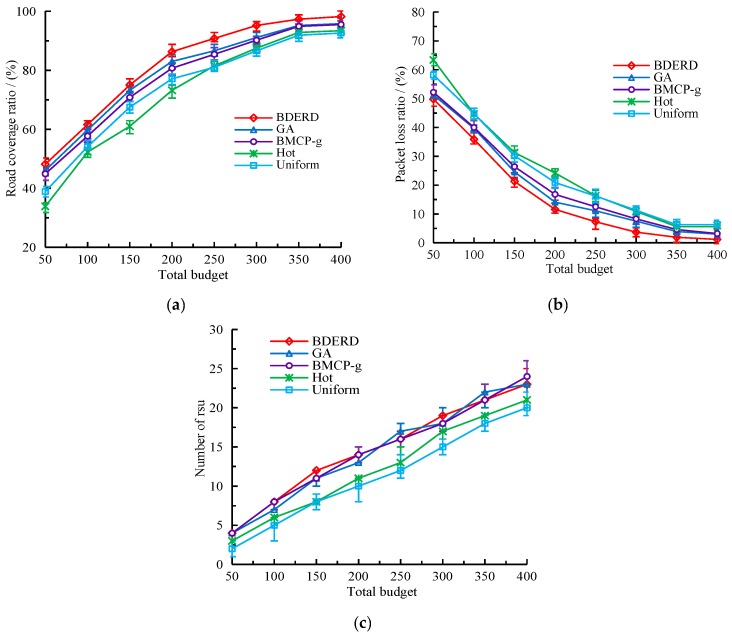
(**a**) Road coverage ratio versus the total budget; (**b**) packet loss ratio versus the total budget; (**c**) number of roadside units (RSUs) versus the total budget.

In this section, we evaluate the performance of different schemes with respect to the total budget. We considered that the deploying area was 8×8, the bounded delay was τ=4s, and that the total budget ranged from 50 to 400, with 50 as a gap. Figure 6a–c illustrate the changes in road coverage ratio, packet loss ratio, and the number of RSUs deployed as the total budget increases. As shown in the figures, when the total budget increases, road coverage ratio and the number of RSUs both rise, while the packet loss ratio decreases. Although the number of RSUs deployed in an area are slightly different when the BDERD, GA, and BMCP-g schemes are adopted, the BDERD scheme outperforms the other four schemes in terms of road coverage ratio and packet loss ratio. This is because the total budget controls the number of RSUs deployed, thus making an increase of covering sub-roads and reducing unsuccessful transmission packets. Moreover, the BDERD scheme performs the greedy-based individual reparation and promotion algorithm, as well as the solution promotion algorithm, to promote the quality of the best solution, meaning it gets the optimal global solution compared to other schemes.

#### 5.2.3. Effect of Deployment Area

**Figure 7:**
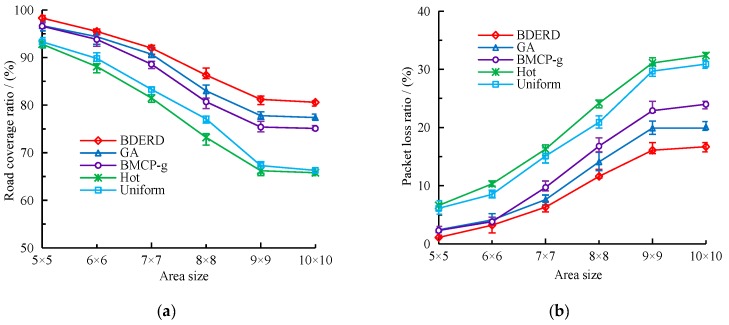
(**a**) Road coverage ratio versus the area size; (**b**) packet loss ratio versus the area size.

So far, we have studied the DBCL problem when the deployment area is fixed. While the size of the area also plays a non-negligible role in RSU deployment, in this section, we outline how we established different areas to assess how the BDERD scheme works when the area changes from 5×5 to 10×10. We set the total budget to 200 and the bounded delay to τ=4s. Figure 7a,b sets out the comparison between the road coverage ratio and packet loss ratio of this scheme to other schemes when the area becomes larger.

As the figures show, enlarging the deployment area can reduce the road coverage ratio and lead to higher packet loss. In this case, the added number of valid covering sub-roads is less than the increasingly divided sub-roads, leading to a decline in road coverage ratio. Although the GA scheme is similar to the BDERD scheme, the traditional genetic algorithm has a precocious feature, it also does not repair unsatisfied solutions nor promote the best solutions it could get after each generation. The approximation greedy algorithm BMCP-g always considers the locations with the most coverage at minimal cost as its best solutions—however, it easily generates local optional solutions. The Hot scheme deploys RSUs based on the greedy algorithm, and neither considers duplicating covering sub-roads nor premeditates its best solutions from a global scene. The Uniform scheme ensures the even distribution of RSUs in the area, but does not take deploying hot-spots into consideration, thus making the best solution it found lack of greediness. Therefore, it can clearly be seen that the performance of these four RSU deployment schemes are lower than our proposed BDERD scheme.

### 5.3. Realistic Simulation

**Figure 8:**
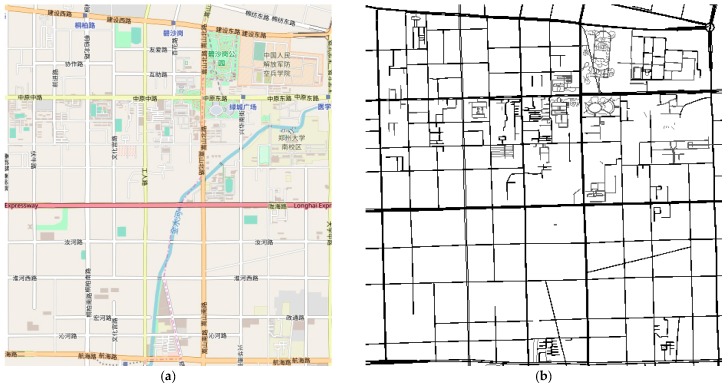
Erqi District, Zhengzhou, China. (**a**) Real map in OSM (Chinese characters in the map just show the name of the roads and area landmarks); (**b**) initial graph in SUMO.

**Figure 9:**
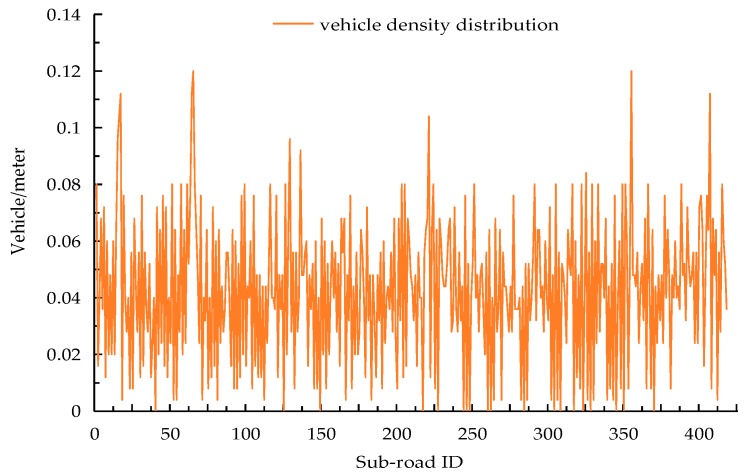
Vehicle density distribution in realistic simulation.

In realistic situations, we used the Simulation of Urban Mobility (SUMO) to convert the real map into a road network. The real map data, from Zhengzhou, China, was obtained through the freely-available OpenStreetMap (OSM). The simulation was written using C++ language (Visual Studio 2015, VS2015) on a Lenovo H3050 personal computer (Inter(R) Core(TM)i5-6400 CPU @ 2.70 GHz, 8 GB RAM, 500 GB Internal HDD, Windows 7 Operating System). Then, we used SUMO to generate VANET realistic simulations, and read the xml files outputted by SUMO to create graphs and vehicle routes in VS2015. Figure 8a shows a real map of the Erqi District in Zhengzhou, China in OSM, and the Chinese characters in Firure 8a just show the name of roads and area landmarks. Figure 8b shows the initial graph in SUMO. The area size is 3.7 km×3.7 km, consisting of 126 intersections and 418 sub-roads. The communication radius is 250 m, and because the distance between the two adjacent intersections did not surpass the communication radius, its length was not changed—if this were not the case, it would have needed to be divided into several sub-roads with their length not exceeding the communication radius. The vehicle density in each sub-road is displayed in Figure 9. In comparison with the density in the synthetic simulation, it is possible that the vehicle density here could be equal to 0 as the lengths of some divided sub-roads between two adjacent intersections may be just a few tens of meters long. Other parameters can refer to their corresponding values in Section 5.2. In this section, we compare the effect of bounded delay and total budget on four different performance metrics.

**Figure 10:**
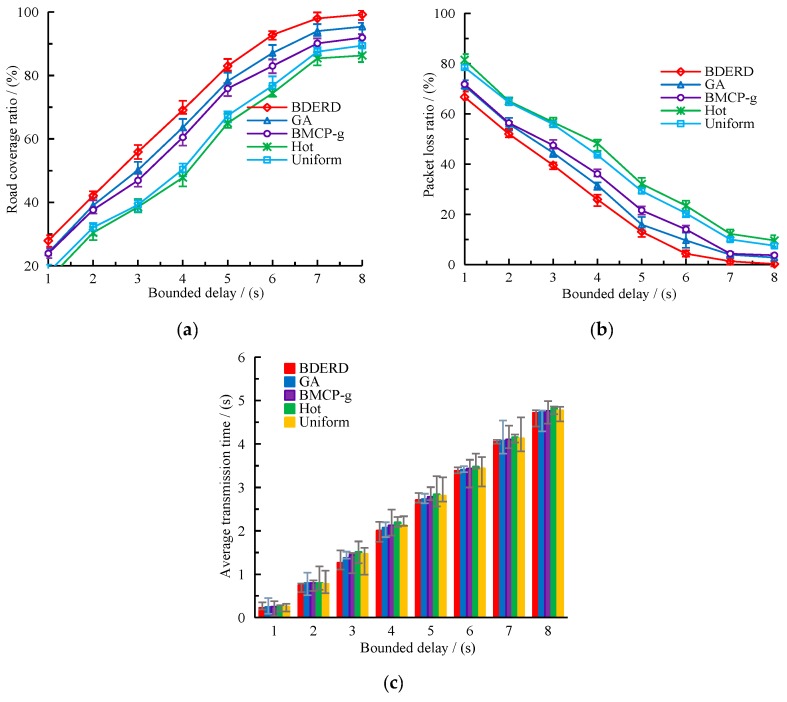
Realistic simulation. (**a**) Road coverage ratio versus bounded delay; (**b**) packet loss ratio versus bounded delay; (**c**) average transmission time versus bounded delay.

Firstly, the impact of bounded delay on the different schemes is analyzed. We set the total budget to 200, and the bounded delay varied from 1 s to 8 s with 1 s as a gap. Figure 10a–c shows the effect of the bounded delay on the road coverage ratio, packet loss ratio, and average transmission time. We can see from Figure 10 that although the three metrics have same change in trend versus the bounded delay in the synthetic situation, they have different values in the given constraint. When the bounded delay is lower than 6 s, the road coverage ratio and packet loss ratio changes fast and become nearly stable after 7 s, while the average transmission time is gradually added. This is because the bounded delay plays a significant role in data transmission, and can quickly increase the number of vehicles which are able to transmit their data to RSUs. It also leads to the average transmission time ups, since a couple of vehicles can use the carry strategy to transmit their data. Also, due to the relatively low vehicle density it has in a realistic road map, the performance of these five deployment schemes is not superior to the synthetic simulation.

**Figure 11:**
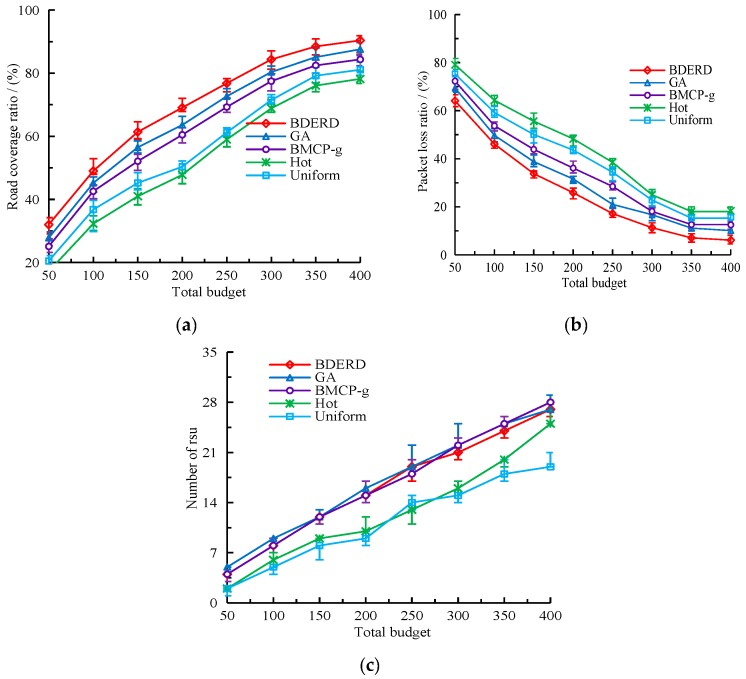
Realistic simulation. (**a**) Road coverage ratio versus the total budget; (**b**) packet loss ratio versus the total budget; (**c**) number of RSUs versus the total budget.

Then, we compared our BEDRD scheme with other deployment schemes when the total budget changed from 50 to 400 and the bounded delay was 4 s. Figure 11a–c presents the changes in road coverage ratio, packet loss ratio, and the number of RSUs. 

It can easily be drawn from the figures that if the total budget is simply increased, the schemes’ performance is not well compared with the increase of bounded delay. When the total budget is within 50 to 300, the road coverage ratio and the packet loss ratio show obvious changes, and then slowly changes with the total budget. The number of RSUs make almost the same alterations when the total budget is added, but will become a fixed value if all the intersections have deployed RSUs.

## 6. Conclusions

In this paper, we studied the delay-bounded and cost-limited RSU deployment problem in urban VANET. We converted the urban road map into a weighted graph and designed a Dijkstra-based algorithm to calculate a 0–1 covering matrix so as to prove that the DBCL problem was NP-hard. After formalizing the DBCL problem into a variation of the 0–1 Knapsack problem, the binary differential evolution-based RSU deployment scheme was proposed. Since the converting 0–1 Knapsack problem is a constrained optimization problem, we used the greedy-based individual reparation and promotion algorithm to change it to an unconstrained optimization problem. Unlike the general differential evolution algorithm, vectors in BDERD are coded in binary integers, and opposite-based learning was used to generate an initial population. A binary differential mutation operator, compliant to binary coding, was also designed, and an improved crossover operator was introduced to increase population diversity. Moreover, the solution promotion algorithm was given to promote the highest-quality vector in each generation. Comparative experiments were done to evaluate the performance of the proposed algorithm. Results show that our proposed algorithm performs better in terms of road coverage ratio and packet loss ratio, with a compromise of bounded delay and a limited budget. For our future work, we will investigate quality-of-service (QOS)-guaranteed RSU deployment in VANET, which will extend the deployment environment to a highway scenery.

## Figures and Tables

**Table 1:** Notations and descriptions.

Notations	Descriptions
R	Communication radius of vehicles and RSUs
C	Budget for RSU deployment
τ	Bounded delay for data transmission
G=(V,E)	$V=\{I_{1},I_{2},\ldots I_{n}\}$ is the set of intersections which are candidate locations for RSUs and satisfies |V|=n, $E=\{E_{1},E_{2},\ldots E_{s}\}$ is the segment set between two intersections
$G_{1}=(V_{1},E_{1})$	$V_{1}=\{V,M\},E_{1}=\{e(i,j),\forall i,j,i\in V_{1},j\in V_{1}\}$, $\left|V_{1}\right|=N,\left|E_{1}\right|=m$ and $M=\{m_{1},m_{2},\ldots m_{k}\}$ Are vertexes formed when dividing into segments E∈G(V,E)
$e(i,j)=\{l_{ij,}\rho_{ij},v_{ij}\}$	$l_{ij}$, $\rho_{ij}$, $v_{ij}$ are the Euclidean distance, vehicle density, and average speed of e(i,j)
t(i,j)	Data transmission time in e(i,j)
$t_{hop}$	One hop transmission time, and $t_{hop}=p_{size}/s$, $p_{size}$ is packet size,sis data rate
T(m×n)	T is a 0–1 covering matrix, if $t_{ji}\leq \pi (i\in [1,m],j\in [1,n])$, $T_{ji}=1$, otherwise $T_{ji}=0$
$t_{j}(1\leq j\leq m)$	Minimum transmission time from vehicles in e(i,j) to RSU
$t_{ji}$	1≤i≤n, minimum transmission time from vehicles in e(i,j) to RSU i∈V
$y_{j}$	if $t_{j}$≤τ, $y_{j}=1$, else $y_{j}=0$
$x_{i}$	=1 if $I_{i}$ is selected to deploy RSU, otherwise $x_{i}=0$
$P_{i}$	τ-covering sub-roads set when $x_{i}=1$
$W_{i}$	Cost for deploying RSU in $I_{i}$

**Table 2:** Results of the binary differential mutation strategy.

$x^{*}\;$	$x^{r1\;}$	$x^{r2\;}$	$v^{i}\;$
0	0	0	0
0	0	1	1
0	1	0	1
0	1	1	1
1	0	0	0
1	0	1	0
1	1	0	0
1	1	1	1
